# Improved Cytotoxic T Lymphocyte Responses to Vaccination with Porcine Reproductive and Respiratory Syndrome Virus in 4-1BB Transgenic Pigs

**DOI:** 10.3389/fimmu.2017.01846

**Published:** 2017-12-18

**Authors:** Guangping Huang, Xianyong Liu, Donal W. Duszynski, Xiaoli Tang, Saeed El-Ashram, Zhengzhu Liu, Xun Suo, Qiuyan Li

**Affiliations:** ^1^State Key Laboratory for Agrobiotechnology, College of Veterinary Medicine, China Agricultural University, Beijing, China; ^2^Key Laboratory of Animal Epidemiology of the Ministry of Agriculture, College of Veterinary Medicine, China Agricultural University, Beijing, China; ^3^Department of Biology, University of New Mexico, Albuquerque, NM, United States; ^4^Department of Animal Science and Veterinary Medicine, Hebei Normal University of Science and Technology, Qinhuangdao, Hebei, China; ^5^State Key Laboratory for Agrobiotechnology, College of Biological Sciences, China Agricultural University, Beijing, China

**Keywords:** immune response, viral infection, 4-1BB, co-stimulation, transgenic pig

## Abstract

Vaccination is the most reliable measure to prevent infectious diseases in domestic animals. Development of novel vaccines demands extensive studies with new technologies, such as using novel adjuvants and immunomodulatory molecules. The co-stimulatory molecule 4-1BB provides a key signal that directs the fate of T cells during activation, and thus is important to their function in immune protection. To determine whether host immune responses to viral infection could be promoted by enhancing 4-1BB co-stimulation, in this study, we produced transgenic pig clones expressing an extra copy of the 4-1BB gene by clustered regularly interspaced short palindromic repeats/CRISPR-associated gene 9-mediated homologous recombination at the *Rosa26* locus. The immune responses of transgenic pigs to porcine reproductive and respiratory syndrome virus (PRRSV) vaccine were determined on day 14. We show that peripheral blood lymphocytes of transgenic pigs expressed around twice the level of 4-1BB mRNA than those of control pigs. We also found IL-2, TNF-α, and granzyme B mRNA levels as well as PRRSV-specific IFN-γ response were significantly upregulated in 4-1BB transgenic pigs, leading to more efficient cytotoxic T lymphocyte (CTL) killing, whereas the expressions of IL-4, IL-17, and Foxp3 were not affected. These results indicate that higher levels of 4-1BB expression involve in promoting Th1 differentiation and enhancing specific CTL responses to PRRSV, and provide a novel approach to increase the efficacy of current vaccines to control the infectious diseases.

## Introduction

Infectious diseases adversely affect livestock production, animal welfare, public health, and the economy (1, 2). Swine have been intensively and extensively produced to meet the growing demand for porcine products (3, 4). However, rapid developments in pig husbandry also increase the risk of infectious diseases spreading from animal to animal as their densities increase. Among the most important maladies are respiratory and enteric diseases (5). Infectious diseases are responsible for huge economic losses in the swine industry worldwide by depressing both daily weight gain and food-conversion efficiency, as well as increasing mortality (6).

Porcine reproductive and respiratory syndrome (PRRS) is one of the most economically devastating diseases that severely threaten swine production worldwide, leading to reproductive failures in pregnant sows and respiratory problems that persistently infect offspring piglets (7, 8). The causative agent of this disease, PRRS virus (PRRSV), is an enveloped, positive-stranded RNA virus, which can undergo rapid evolution and present as a genetically and antigenically heterogeneous population (8). The virus has a very restricted tropism for cells of the monocytic lineage, and the fully differentiated porcine alveolar macrophage serves as a primary cell target for PRRSV infection (9). CD163, as a member of the scavenger receptor cysteine-rich family, has been identified as the key receptor that mediates viral internalization and disassembly (10, 11). To make things worse, PRRSV induces an inadequate T cell response and likely poor quality of cytotoxic T lymphocyte (CTL) response (12, 13), which may cause secondary or opportunistic pathogens infection, leading to more serious and chronic diseases.

Vaccination is a feasible and low-cost strategy and has been widely adopted to reduce PRRSV-associated economic losses. Compared with the killed virus vaccines that are generally less efficacious, PRRS-modified live virus vaccine is more effective and has been extensively used worldwide (14). A strong T cell-mediated immune response plays a central role in eliminating intracellular pathogens like PRRSV (15–17). However, PRRSV-specific T cells do not appear until 2 weeks after vaccine inoculation, and T cell immune response is transient in all cases (18). Therefore, upregulating the host immune responses to commercial vaccines will be a promising strategy for the prevention of infectious diseases.

For optimal activation of naive T cells, signals delivered *via* the binding of co-stimulatory molecules to their ligands are essential, as antigen-activation of T cells without co-stimulation may lead to T cell anergy (19). There have been many developments with the co-stimulatory and co-inhibitory molecules, including CD28, CTLA4, and PD-1, and targeting T cell co-signaling is now a promising approach in the field of immunotherapy (20, 21). Another example of T cell co-stimulation is provided by the membrane-spanning glycoprotein (4-1BB), a member of the tumor necrosis factor receptor superfamily, which is expressed within 24 h of CD4^+^ and CD8^+^ T cell activation (22, 23). When engaged with its ligand (4-1BBL) or with an antagonist antibody, 4-1BB provides a unique co-stimulatory signal late in the primary response after CD28 downregulation that promotes T cell survival by upregulating antiapoptotic factors (24). In addition, co-stimulation through 4-1BB activates AKT to promote cell cycling and IL-2 production (24).

The role of 4-1BB-mediated co-stimulation signaling has been characterized in both mouse and human that leads to the expansion of CD4^+^ and CD8^+^ T cells, production of cytokines, development of CTL effector function, initiation of antiviral and antitumor T cell responses, and high frequency of memory CD8^+^ T cells (25–27). When encountering the recall antigens, 4-1BB provides an important signal to promote memory CD8^+^ T cell proliferation and induce memory CTLs response, suggesting its potential to improve vaccines for the elimination of intracellular pathogens (24). However, its potential for enhancing immune responses in pigs is still unknown.

Recently, clustered regularly interspaced short palindromic repeats (CRISPR), in combination with the CRISPR-associated gene 9 (Cas9) system, has facilitated both insertions/deletions and specifically targeted mutagenesis in mammalian genomes (28). With the development of methods in reproductive biology and gene transfer, the ability to edit (delete, insert, and modify DNA sequences) the porcine genome has opened a new era for breeding of swine varieties. CRISPR/Cas9, combined with transgenic (Tg) technology, offers the possibility to site-specifically introduce gene sequences to obtain a desired phenotype (29–33). To test our hypothesis that host immune responses to viral infection can be enhanced by elevating the level of 4-1BB mRNA, we produced Tg pig clones expressing an extra copy of the 4-1BB gene and characterized their response to PRRSV vaccination, as well as the response to *in vitro* stimulation in this study. As we show next, 4-1BB signaling is indispensable for Th1 differentiation, and CTL effector function.

## Materials and Methods

### Ethics Statements

All animal experiments in this study were performed in strict accordance with the Guide for the Care and Use of Laboratory Animals of the Ministry of Science and Technology of China and were approved by the Institutional Animal Care and Use Committee of China Agricultural University (certificate of the Beijing Laboratory Animal employee, ID: 18086). All efforts were made to minimize animal suffering.

### Construction of the 4-1BB Gene Knockin Vector for *Rosa26* Targeting

**Figure 1:**
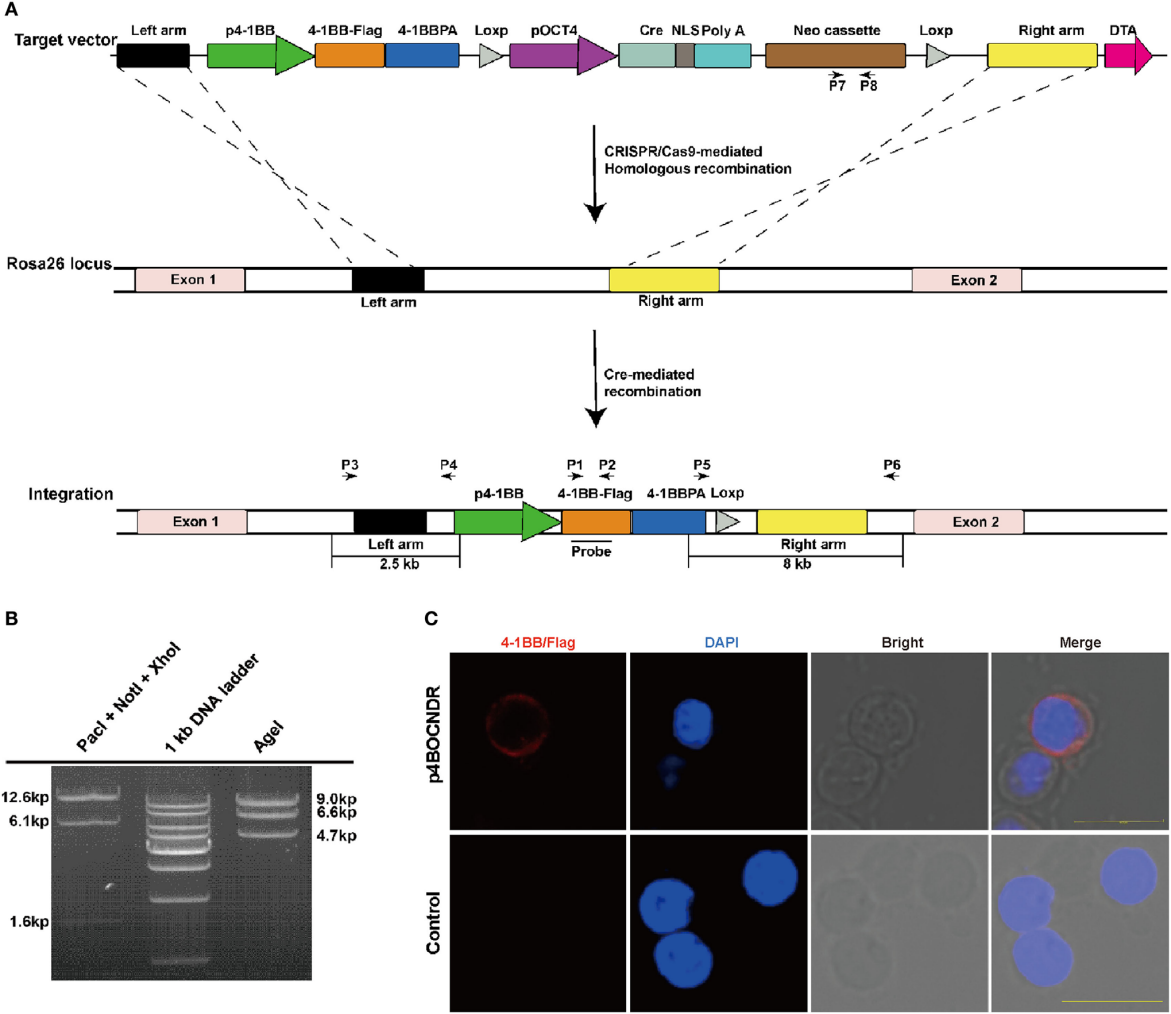
Construction and detection of the 4-1BB gene knockin vector for *Rosa26* targeting. **(A)** A schematic diagram of genome editing strategy to target the *Rosa26* locus in *Sus scrofa via* clustered regularly interspaced short palindromic repeats (CRISPR)/CRISPR-associated gene 9 (Cas9)-mediated homologous recombination and *Cre*-mediated recombination. The black arrows indicate the primers used for PCR assay. p4-1BB, 4-1BB promoter; 4-1BB, 4-1BB coding sequence; 4-1BBPA, 4-1BB 3′ untranslated region; pOCT4, OCT4 promoter; NLS, nuclear localization sequence; Neo, neomycin resistance gene; DTA, diphtheria toxin A cassette. **(B)** Restriction enzyme digestion analysis of plasmid p4BOCNDR. **(C)** Representative immunofluorescent images of peripheral blood lymphocytes with p4BOCNDR showing the expression of 4-1BB-FLAG on the cell membrane of lymphocytes at 24 h post-transfection. Scale bar, 10 µm.

The *Rosa26* locus, which produces three non-coding transcripts of unknown function (34, 35), was used as the target site for stable integration of the duplicate 4-1BB gene in pigs. The donor plasmid p4BOCNDR was constructed according to the following strategy. First, a 4-1BB expression cassette containing the coding sequence (CDS) of this gene (GenBank No. JQ663443) and its 5′ and 3′ regulatory elements were constructed. The FLAG-tag sequence was 24 bp in size and was added to the c-terminus of the 4-1BB CDS by PCR amplification. Second, the Oct-4 promoter, which is specifically activated during embryonic development (36), was cloned into a site upstream of the *Cre* recombinase gene. Third, two loxP sequences with the same orientation were ligated to the 5′ end of the *Cre* expression cassette and the 3′ end of the *neo* expression cassette, respectively. Fourth, 2- and 6-kb fragments of the first intron of *Rosa26* were amplified and used as left- and right-homologous arms, respectively. Finally, the left-homologous arm, 4-1BB expression cassette, *Cre* expression cassette (with the 5′ loxP sequence), *neo* expression cassette (with the 3′ loxP sequence), right-homologous arm and negative selection marker diphtheria toxin A (DTA) expression cassettes were ligated together to produce donor plasmid p4BOCNDR (Figure 1A). p4BOCNDR was validated by restriction enzyme digestion and sequencing.

### Detection of 4-1BB Expression in Host Cells Transfected with p4BOCNDR *In Vitro*

Peripheral blood mononuclear cell (PBMC) was isolated from a 2-month-old pig. Venous blood was collected in 5 mM anticoagulant EDTA (final concentration) and centrifuged on discontinuous Percoll gradients (Sigma, St. Louis, MO, USA) as previously described (37). PBMCs were >98% viable as confirmed by Trypan blue (Sigma) exclusion. PBMCs were seeded at a density of 1 × 10^6^ cells/mL of RPMI-1640 medium (Gibco, Carlsbad, CA, USA) supplemented with 10% heat-inactivated fetal bovine serum, 20 mM HEPES, 2 mM glutamine, and antibiotics (100 U/mL penicillin and 100 µg/mL streptomycin) at 37°C in a humidified atmosphere containing 5% CO_2_.

Peripheral blood mononuclear cells were nucleofected with p4BOCNDR plasmid using Amaxa Nucleofector (Lonza Group AG Basel, Switzerland), following the instruction of the manufacturer. Transfected cells were stimulated using concanavalin A (ConA, 2 µg/mL) and incubated at 37°C with 5% CO_2_. Cells were harvested at 24 h post-nucleofection. Indirect immunofluorescence assay (IFA) was performed following the standard protocol. Briefly, permeabilized and blocked cells were incubated with anti-FLAG monoclonal antibody (F1804, Sigma-Aldrich) and then with the goat anti-mouse IgG H&L (Cy3) secondary antibody (ab97035, Abcam, Cambridge, UK). Nuclei of cells were stained with 4′,6-diamidino-2-phenylindole (Sigma-Aldrich), and then slides were observed under a fluorescence microscope (Leica TCS SP5, Leica Microsystems, Germany).

### Construction of pCas9-sgRNA and *In Vitro* Testing of Its Cleavage Efficiency

sgRNAs targeting intron 1 of *Rosa26* were designed using the CRISPRtool (http://crispr.mit.edu) to minimize off-target effects. The annealed oligonucleotides were inserted into the backbone of plasmid pX330 (CBh::SpCas9 + U6::chimeric guide RNA, 42230, Addgene) to construct pCas9-sgRNA as previously described (38). The cleavage efficiency of Cas9 at the targeting site was tested using a T7 endonuclease I (T7EI) assay. Briefly, porcine fetal fibroblasts (PFFs) were transfected with pCas9-sgRNA. Genomic DNA extracted 48 h post-transfection was subjected to PCR with primers P9 and P10. PCR products were purified and then digested with T7EI and electrophoresed on a native polyacrylamide gel. Gel images were analyzed using ImageJ 1.37 software as previously described (39). PCR products were sequenced to detect mutations within intron 1 of *Rosa26*.

### Screening of Positive Tg Cell Colonies and Generation of 4-1BB Tg Pigs by Nuclear Transfer

Primary PFFs were isolated and cultured as described previously (40). Then, 1 × 10^6^ PFFs were co-transfected with 4 µg of linearized p4BOCNDR and 1 µg of pCas9-sgRNA and seeded onto 10 cm^2^ dishes. After 10 days of positive selection with G418 (with the concentrations ranging from 500 to 1,000 µg/mL) and negative selection with DTA, cell clones visible by the naked eyes were isolated with cloning cylinders, trypsinized, and transferred to 24-well plates. Subcloned cells were cultured for another 48 h before transfer to a 6-well plate. One-fourth of the cells from each well of the 24-well plate were collected and used for PCR analysis using two primer pairs (P3/P4 and P5/P6). Cells from each correctly modified clone were cryopreserved in liquid nitrogen and used as donor cells for somatic cell nuclear transfer (SCNT). SCNT was conducted as described previously (41). Briefly, the nucleus of an unfertilized, haploid egg cell was extracted and replaced by a nucleus from a diploid donor cell. The reconstructed zygotes were treated *in vitro* with electrical fusion and chemical activation. Three hundred reconstructed embryos were transferred into both oviducts of each surrogate on day 1 of the onset of estrus. Pregnancy was determined by abdominal ultrasound examination 1 month after SCNT. Piglets were delivered by natural birth after approximately 114 days of gestation and housed in BSL-2 conditions at Taichang Agriculture and Animal Husbandry Co., Ltd. (Qinhuangdao, Hebei Province, China).

### Identification of Tg Pigs

To detect the integration of 4-1BB into the *Rosa26* locus, 10 µg of genomic DNA extracted from the tail samples of Tg or wild-type (WT) pigs were digested with *Not*I overnight and then resolved by 1% agarose gel electrophoresis. DNA samples were transferred from the agarose gel onto a nylon membrane and then hybridized with a digoxigenin (DIG)-labeled probe for amplification with primers P1 and P2 as indicated in Figure 1A. Then, the samples were incubated with anti-DIG AP (Roche), and the location of the probe was detected by chemiluminescent methods.

To detect integration of the 4-1BB expression cassette into the *Rosa26* site, genomic DNA and four primer pairs (P1–P8, Table 1) were used to amplify the 4-1BB CDS, 5′ and 3′ homologous arms and *neo* gene by PCR.

**Table 1:** Primer pairs used for conventional PCR.

Primer	Sequences(5′–3′)	Amplicon size (bp)
P1	GGCTACTACAACATAGTGGC	502
P2	AAGTGTGACCTGGAGAGAAG	
P3	CGCACCCTTACCTTGTCCCA	2,500
P4	GAAGGTGGGATGGAGGGTGA	
P5	CCTTATGTTGGGTTGGCATC	8,073
P6	AGCTGCCTCCTGTGATTACC	
P7	CTATGACTGGGCACAACAGACAAT	677
P8	CGATACCGTAAAGCACGAGGAA	
P9	ATCTTGAGCATAGGCCCAAC	282
P10	GACAGAGACACTAGTTTGG	

To detect transcription of 4-1BB, the total RNA was extracted from PBMCs of Tg or WT pigs post-stimulation using TRIzol reagent according to manufacturer instructions (Invitrogen, Carlsbad, CA, USA). All RNA samples were treated with DNAse before cDNA synthesis. Total RNA was reverse-transcripted using an EasyScript First-Strand cDNA Synthesis SuperMix (Transgen, Beijing, China). Primers for real-time PCR are shown in Table 2. For PCR amplifications, 1 µL of cDNA was added to a mixture containing 10 µL of 2× SYBR Green Master Mix (Takara, Dalian, China), 0.4 µL of ROX reference dye, and 0.4 µL of each primer (50 pmol/μL). Three transgenic and five wild-type pigs were used to analyze the relative mRNA levels between the two groups with the 2^−ΔΔCT^ method (42). The mean ΔCT of five wild-type samples was used as the control (set to 1). The ΔΔCT was calculated by comparing the ΔCT of three transgenic pigs (*n* = 3) with controls. Transcripts of porcine GAPDH were used as an internal reference. All samples were tested in duplicate.

**Table 2:** Primer pairs used for quantitative real-time PCR.

Target gene	Forward and reverse primer sequences^a^ (5′–3′)
IL-2	F: TAGTAGAAGAACTCAAAGCTCTGG
	R: CTTGTTTCAGATCCCTTTAGTTCC
IL-4	F: GGCAAACATGACCTGTTCTG
	R: CCTTCATAATCGTCTTTAGCCT
IFN-γ	F: GCAAGTACCTCAGATGTACCT
	R: TTGTCACTCTCCTCTTTCCA
TNF-α	F: CCCAGAAGGAAGAGTTTCCA
	R: TTTGACATTGGCTACAACGTG
4-1BB	F: GTCATCATCTTCTTTCTTGCAC
	R: CTTCAGAAACGGTTGTTTGAC
Granzyme B	F: CCTACATGGCGTATCTTCAG
	R: GTGACGTTGATTGAGCTTCC
GAPDH	F: CTCAACGGGAAGCTCACTGG
	R: TGATGTCATCATATTTTGCAGGTT
IL-17	F: CTCTCGTGAAGGCGGGAATC
	R: GTAATCTGAGGGCCGTCTGG
Foxp3	F: CCCTGCCCTTCTCATCCA
	R: GTGGCCCGGATGTGAAAA

*^a^These primers were designed by Zhao et al. (43)*.

To measure expression levels, PBMCs, stimulated with ConA (2 µg/mL), were cultured for 24 h, lysed with RIPA buffer (10 mM Tris, pH 7.4; 150 mM NaCl; 0.2% Triton X-100; 2 mM EDTA; 1 mM PMSF; and 1× Protease Inhibitor Mixture), and then separated on 15% polyacrylamide gels. Separated proteins were transferred to nitrocellulose membranes (Amersham Pharmacia, UK) that were blocked with 5% skim milk in phosphate-buffered saline Tween 20 (PBST) at 25°C for 1 h, and then detected using anti-FLAG mouse monoclonal antibody (F1804, Sigma-Aldrich). The proteins were then incubated with goat anti-mouse IgG-HRP (ab97023, Abcam) for 1 h at 25°C, followed by washing three times with PBST. The membranes were subjected to luminol-based chemiluminescence detection using a commercial substrate (Millipore, USA) and Kodak film. IFA was also performed on PBMCs as described earlier.

### Flow Cytometry Analysis

For T cell proliferation analysis, PBMCs from Tg and WT pigs were labeled with carboxyfluorescein diacetate succinimidyl ester (CFSE) dye (BioLegend) as previously described (44). Briefly, cells were incubated with 5 µM CFSE in PBS containing 5% FBS at 37°C for 5 min and washed three times with ice-cold PBS-5% FBS. 2 × 10^5^ CFSE-labeled PBMCs/well of a 96-well plate were cultured in RPMI-1640 media supplemented with 10% heat-inactivated FBS, 1% penicillin/streptomycin in the presence of stimulation. PBMCs were harvested after a 3-day culture for subsequent analysis by the BD FACSVerse™ flow cytometer.

Peripheral blood mononuclear cells were cultured in 24-well round bottom plates (1 × 10^6^ cells/well) and stimulated for 0, 24, 48, and 72 h with ConA (2 µg/mL), recovered by centrifugation and re-suspended in 100 µL of PBS containing 10% porcine serum. After being washed twice with a cell-staining buffer (BD Biosciences), the cells were suspended in 50 µL of staining buffer and stained with mouse anti-pig 4-1BB monoclonal antibody at 4°C for 30 min. After two washes, they were stained with FITC anti-mouse IgG (BioLegend Cat. No. 406001). Then, the cells were washed two times, suspended in 200 µL of sterile PBS, and analyzed using the BD FACSVerse™ flow cytometer.

### Detection of Cytokine and Cytolytic Mediator Production Postvaccination with PRRSV

Both 4-1BB Tg and WT pigs were inoculated intramuscularly with a modified live PRRSV vaccine (JXA1-R, Qilu Animal Health Products Co., Ltd., Jinan, China) at 14 days of age. PBMCs, sampled 2 weeks postvaccination, were used to determine the expression levels of cytokines and a cytolytic mediator. The expression of Th1- (IL-2 and IFN-γ) and Th2-type (IL-4) cytokines were determined by real-time PCR. Furthermore, PBMCs were stimulated *in vitro* for 48 h with the PRRSV vaccine strain (2 × 10^5^ TCID_50_, propagated by three rounds of plaque purification in MARC-145 cells) and then the production of Th1- (IL-2, IFN-γ, and TNF-α), Th2- (IL-4), Th17-type (IL-17) cytokines, transcription factor (Foxp3) of regulatory T cell and a cytolytic mediator (granzyme B) were measured.

The concentrations of IL-2, IFN-γ, and IL-4 in serum were measured using commercial enzyme-linked immunosorbent assay (ELISA) kits (BlueGene Biotech, Shanghai, China) at day 14 postvaccination. All ELISA procedures were performed as per the manufacturer’s instructions. Each sample was assayed in triplicate.

### Enzyme-Linked ImmunoSpot (ELISPOT) Assay

Interferon-γ ELISPOT was performed as previously described (45). Briefly, 96 wells of ELISPOT plates were coated with anti-porcine IFN-γ monoclonal antibody (5 µg/mL) (Mabtech, Mariemont, OH, USA). 5 × 10^5^ PBMCs from PRRSV-vaccinated three Tg and three WT pigs in 100 µL RPMI-1640 complete medium were added in each well. Cells were stimulated with PRRSV strain JXA1-R (2 × 10^5^ TCID_50_) while the wells that received an equal amount of bovine serum albumin-coated beads or 2 µg/mL ConA were used as negative and positive controls, respectively. Cells were cultured for 36 h at 37°C in 5% CO_2_. The plates were then washed five times with PBS (200 µL/well). Captured IFN-γ was detected with biotinylated anti-porcine IFN-γ antibody P2C11 and streptavidin–ALP. All procedures were conducted according to the instructions of the manufacturer (Mabtech, Mariemont, OH, USA).

### CTL Assay

To evaluate the ability of upregulated 4-1BB signaling to enhance the antiviral cellular immune responses, a CTL assay was performed with porcine alveolar macrophages as target cells that were infected with PRRSV (JXA-1R strain, multiplicity of infection = 10) and PBMCs from PRRSV-vaccinated pigs restimulated *in vitro* with PRRSV for 7 days as a source of PRRSV-specific CTLs. The viability of target cells following treatment was assessed using an MTT Cell Proliferation and Cytotoxicity Assay Kit following the manufacturer’s instruction (Beyotime Biotechnology, China).

### Statistical Analysis

All experimental data were analyzed using SPSS software (V. 19.0; SPSS Inc., Chicago, IL, USA). Data were expressed as mean ± SEM. Statistical significance was determined using a two-tailed Student’s unpaired *t*-test or one-way ANOVA, and *P* values less than 0.05 were considered significantly different.

## Results

### Construction and Characterization of 4-1BB Knockin Vector p4BOCNDR

The size of vector p4BOCNDR is 20.3 kb (Figure 1A). As expected, digestion with *Pac*I, *Not*I, and *Xho*I fragmented the vector into three bands of 12.6, 6.1, and 1.6 kb, respectively, whereas digestion with *Age*I produced three fragments of 9.0, 6.6, and 4.7 kb (Figure 1B). Expression of the cDNA copy of 4-1BB was confirmed by staining with monoclonal antibody against FLAG tag, which was fused in-frame to 4-1BB on the surface of transfected PBMCs (Figure 1C).

### Detection of the Cleavage Efficiency of CRISPR/Cas9 at the *Rosa26* Locus

The sgRNA (5′-ATCTTGACTACCACTGCGAT-3′) targeting the *Rosa26* locus with the best rank in CRISPRtool was tested for cutting efficiency. Two cleaved bands were generated after the T7EI assay, indicating that our pCas9-gRNA was efficient in generating mutations at the *Rosa26* locus. Out of 20 sequenced clones, 4 had deletion or insertion of random nucleotide(s), confirming efficient cleavage at this site. Interestingly, a nucleotide deletion of up to 31 bp occurred downstream of the target site (data not shown).

### CRISPR/Cas9-Mediated Site-Specific 4-1BB Insertion in Pig PFFs

**Figure 2:**
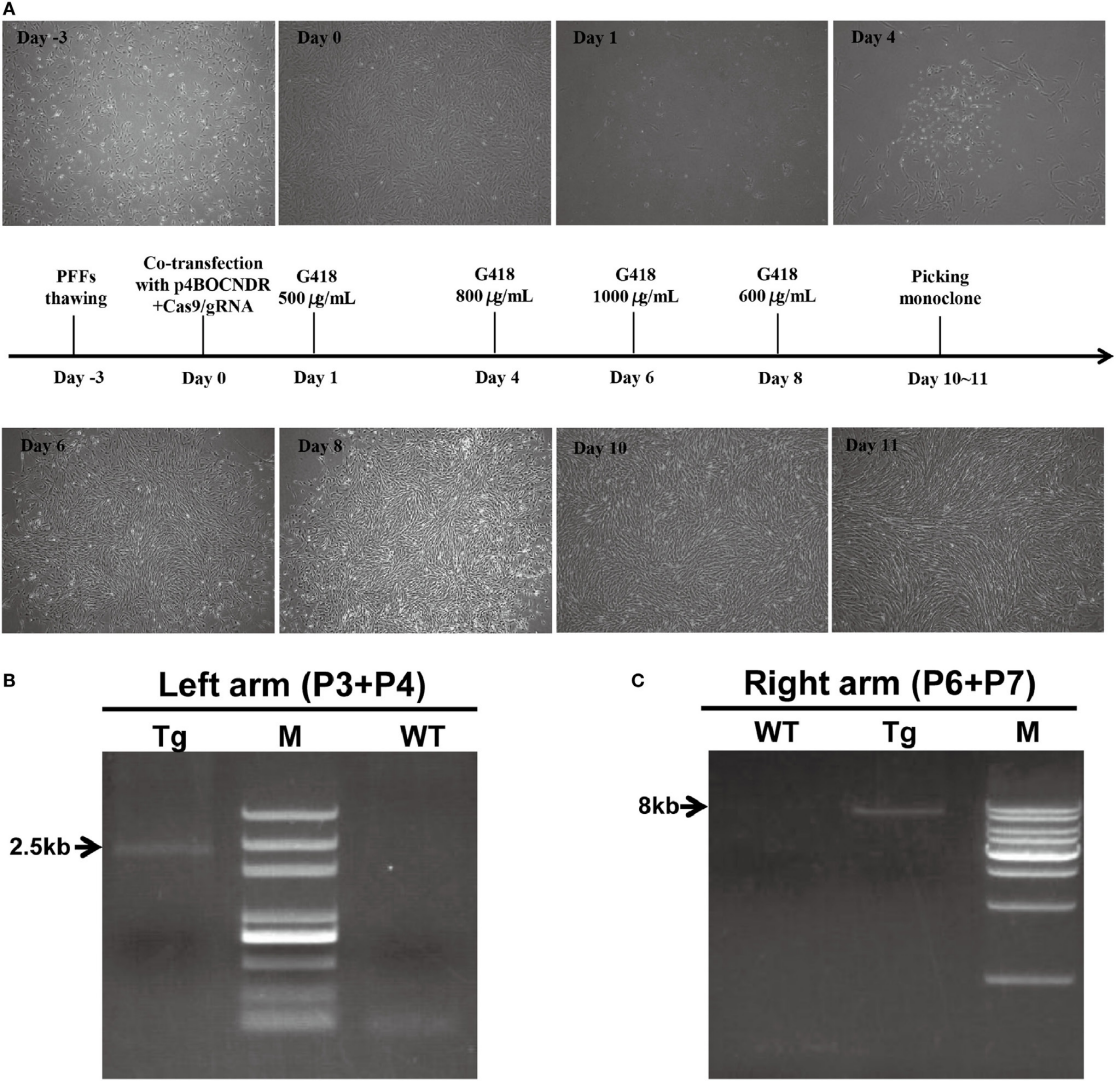
Clustered regularly interspaced short palindromic repeats/CRISPR-associated gene 9-mediated site-specific 4-1BB insertion in pig porcine fetal fibroblasts (PFFs). **(A)** Timeline of the PFF screening process. **(B,C)** Results of the PCR analysis of transgenic clones. The DNA markers (M) indicated in **(B,C)** were D2000 plus and a 1-kb DNA ladder, respectively. Tg, transgenic clone; WT, wild-type.

As a consequence of G418 selection, an obvious reduction in non-transfected cells and the visible formation of living cell foci were observed (Figure 2A). Cell clones with integration and expression of the 4-1BB gene yielded PCR products of 2.5 kb for the 5′ fragment and 8 kb for the 3′ fragment as expected (Figures 2B,C). PCR products were then sequenced to confirm targeting. In total, 10 out of 48 drug-resistant clones showed the expected recombination post co-transfection with p4BOCNDR and Cas9/gRNA, with the transgene inserted specifically at the *Rosa26* locus, whereas 2 out of 19 clones with the single plasmid p4BOCNDR showed the correct recombination. The efficiency of homologous recombination increased strikingly with CRISPR/Cas9-mediated editing.

### Integration and Expression of the 4-1BB Gene at the *Rosa26* Site Was Detected in Tg Pigs

**Figure 3:**
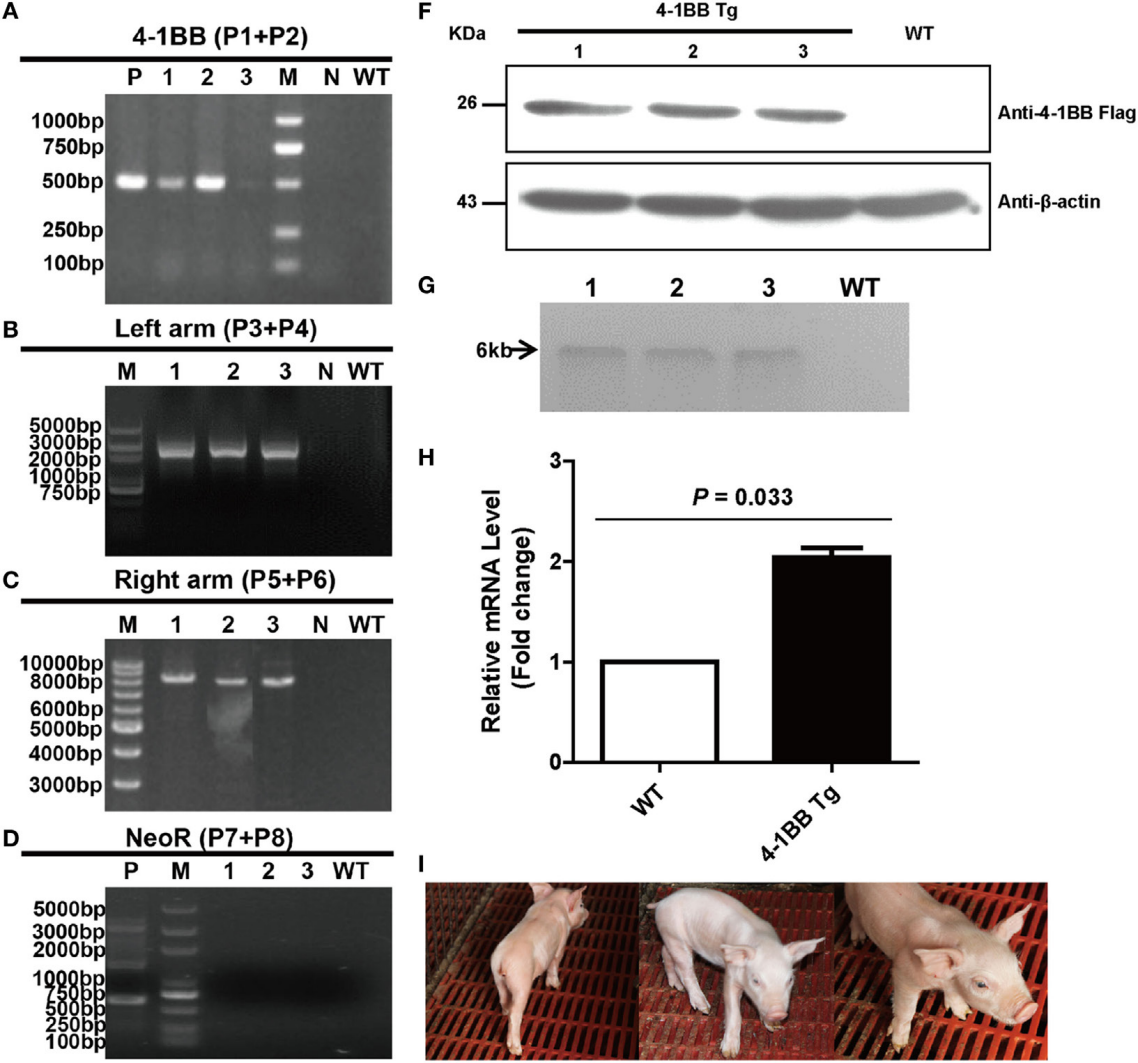

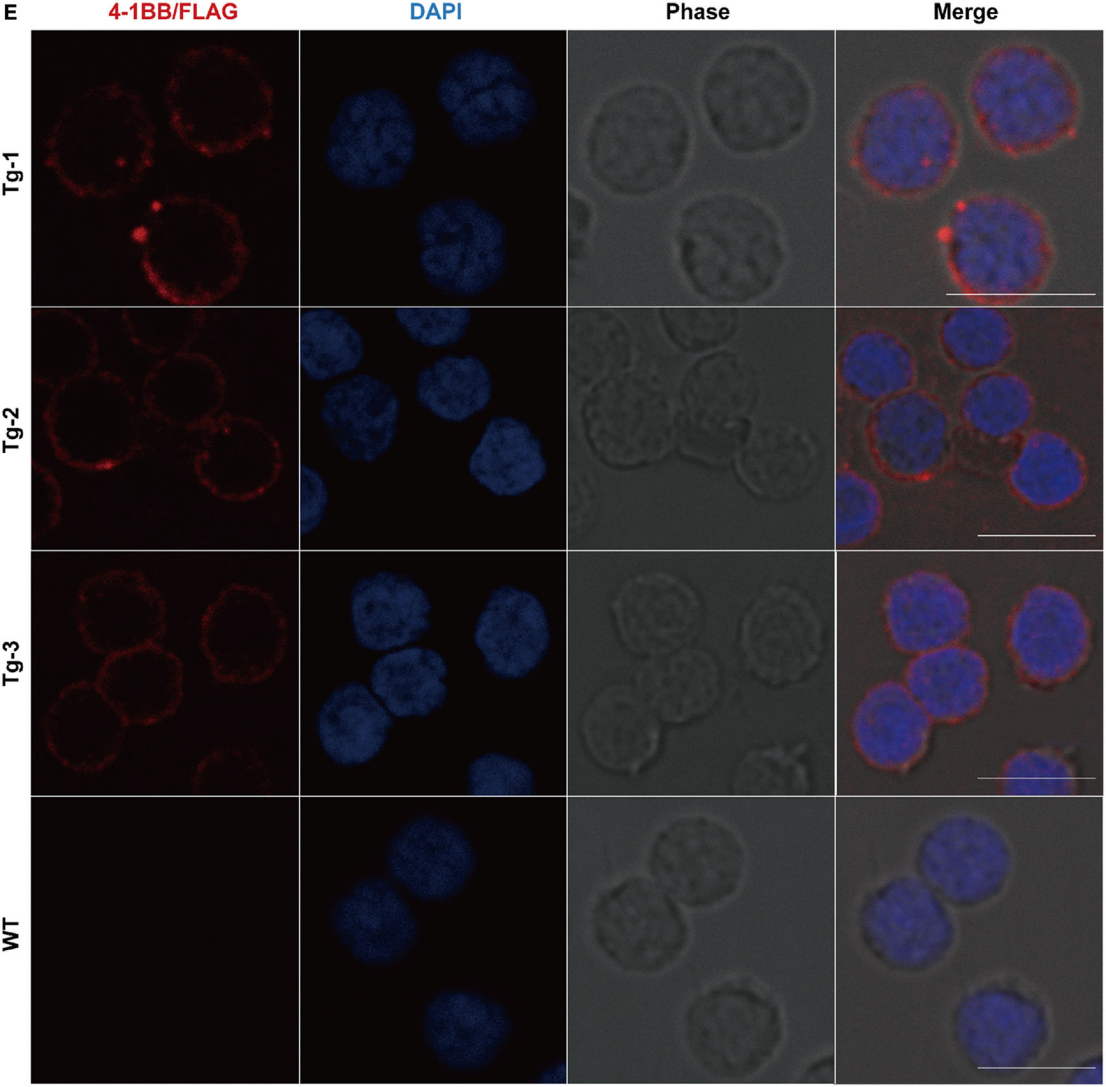
Production and identification of 4-1BB transgenic pigs. **(A–D)** PCR analysis of transgenic piglets using the indicated primers. 1–3, Genomic DNA from transgenic piglets; N, water; WT, wild-type pig. P in panel **(A)** indicates plasmid p4BOCNDR used as positive control. P in panel **(D)** indicates transgenic fibroblasts used as a positive control. **(E)** Representative immunofluorescent images of peripheral blood lymphocytes from transgenic pigs showing the expression of 4-1BB-FLAG on the cell membrane of lymphocytes. Scale bar, 10 µm. **(F)** Western blot analysis of peripheral blood lymphocytes from transgenic pigs. WT, peripheral blood lymphocytes from wild-type pig. **(G)** Southern blot analysis of transgenic pigs. WT, genomic DNA from wild-type pigs. **(H)** Real-time PCR analysis of the 4-1BB mRNA levels in transgenic (4-1BB Tg) and wild-type pigs (WT). *P* < 0.05 was considered statistically significant. **(I)** Images of F0 4-1BB transgenic pigs.

A correctly targeted PFF cell line obtained by selection was used as a donor cell line for SCNT. One out of five surrogate mothers became pregnant, and three cloned piglets were born 114 days later. PCR results showed that the sizes of amplicons were 0.5 kb with primers P1 and P2, 2.5 kb with primers P3 and P4, 8 kb with primers P5 and P6, and 0.7 kb with primers P7 and P8 (Figures 3A–D). As expected, CRISPR/Cas9-mediated site-specific homology recombination occurred at the *Rosa26* locus. DTA, as the counter-selection marker, further improved the odds of successful recombination. The neomycin gene was removed *in vivo* following the expression of *Cre*, regulated by the Oct4 promoter, during early embryogenesis. Site-specific insertion was also confirmed by Southern blot analysis, with a single 6-kb DNA band indicating correct targeting (Figure 3G). To verify efficient expression of 4-1BB at the *Rosa26* locus in the cloned piglets, IFA and western blot were also performed as described earlier. Expression of 4-1BB-FLAG was detected in PBMCs from all three piglets (Figures 3E,F). The results of real-time PCR showed that the levels of 4-1BB mRNA in Tg pigs were twofold higher than those in WT pigs (*P* = 0.033, Figure 3H). It should be noted that all Tg piglets developed normally, and they showed no significant histological abnormalities (Figure 3I).

### Overexpression of 4-1BB Promotes the Proliferation of Peripheral Blood Lymphocytes

**Figure 4:**
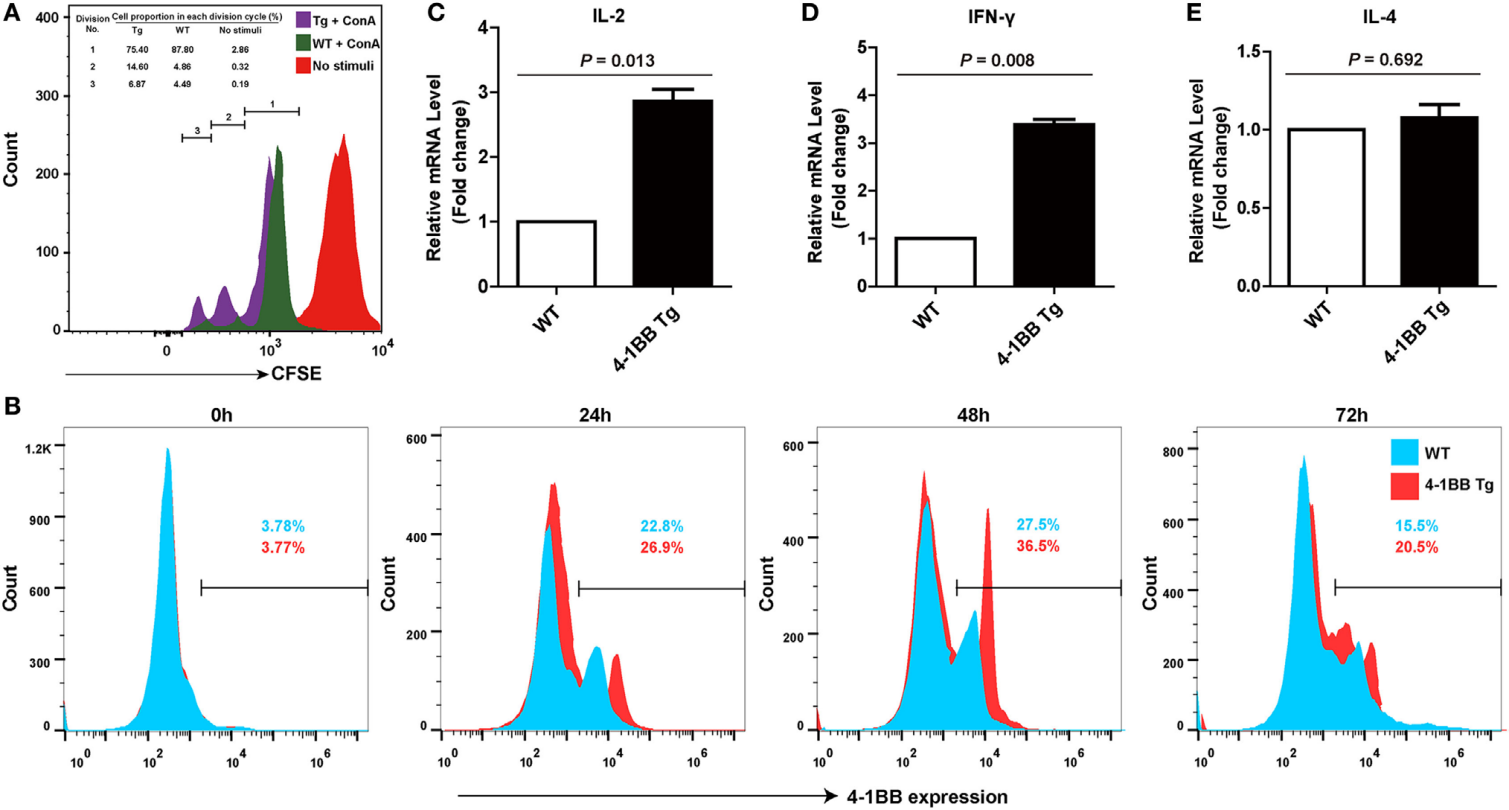
4-1BB overexpression promotes the proliferation of peripheral blood lymphocytes and the levels of Th1 cytokines mRNA expression. **(A)** Equal numbers of peripheral blood lymphocytes were labeled with carboxyfluorescein diacetate succinimidyl ester and stimulated with concanavalin A. Representative results of an *in vitro* proliferation assay showed that 4-1BB transgenic pigs had a greater proliferation capacity compared with pigs of wild-type control. The percentages of cells in each division cycle compared with non-stimulated control (complete RPMI media) were indicated. **(B)** Kinetics of 4-1BB expression on *in vitro* activated T cells. Peripheral blood lymphocytes were stained for 4-1BB expression after stimulation with ConA at indicated times. **(C–E)**: IL-2 **(C)**, IFN-γ **(D)**, but not IL-4 **(E)** mRNA expression in peripheral blood lymphocytes were determined by quantitative real-time PCR during the primary response to porcine reproductive and respiratory syndrome virus vaccination. 4-1BB Tg, 4-1BB transgenic pigs; WT, wild-type pigs as a control. The results shown are representative of three independent experiments. Data are presented as mean values ± SEM. *P* < 0.05 was considered statistically significant.

To evaluate the proliferation capacity of 4-1BB transgenic T cells, we performed an *in vitro* proliferation assay. A CFSE division profile of 4-1BB transgenic T cells during 3 days showed more rapid cell division than WT control, and the percentage of cells in each division cycle compared with non-stimulated control was shown in Figure 4A. This revealed that T cells from 4-1BB transgenic pigs had greater proliferation capacity than those from WT control, consistent with the 4-1BB-deficient mouse phenotype showing impaired T cell proliferation (27). Flow cytometry analysis of 4-1BB expression determined that naïve T cells did not express detectable levels of 4-1BB in both groups. There was a profound increase in 4-1BB expression until 48 h, and by 72 h levels of 4-1BB decreased (Figure 4B).

### Overexpression of 4-1BB Amplifies Th1 Cytokine Secretion in the Immune Response to Vaccination with Live PRRSV Strain

To assess the role of the 4-1BB co-stimulatory pathway on the immune responses to PRRSV, Th1- (IL-2 and IFN-γ) and Th2-type (IL-4) cytokines were measured. Higher expression levels of IL-2 (*P* = 0.013) were observed in 4-1BB Tg pigs, relative to those in WT controls (Figure 4C). However, the remarkable increase in IFN-γ production (*P* = 0.008) and the slight but no significant change in IL-4 production (*P* = 0.692) in Tg pigs (Figures 4D,E) suggested that the 4-1BB signal enhances the induction of immunity by promoting a Th1-dominant response. There was no significant difference in the titer of anti-PRRSV antibodies between Tg and WT pigs (data not shown).

### Overexpression of 4-1BB Enhances Specific CTL Immune Responses

**Figure 5:**
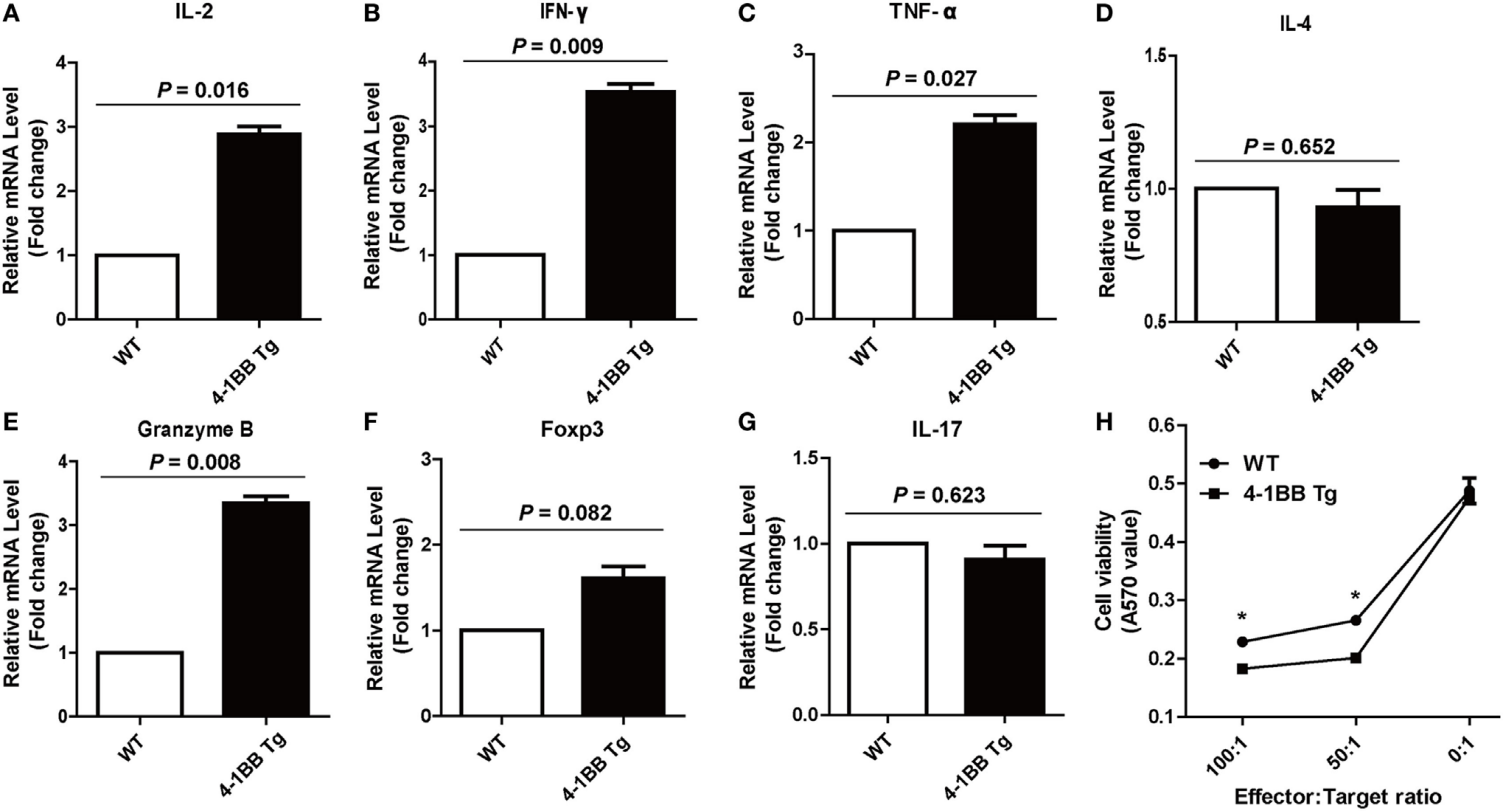
4-1BB overexpression enhances specific Th1 cytokines production and cytotoxic T lymphocyte (CTL) function during the recall response. Levels of IL-2 **(A)**, IFN-γ **(B)**, TNF-α **(C)**, IL-4 **(D)**, granzyme B **(E)**, Foxp3 **(F)**, and IL-17 **(G)** mRNA expression in peripheral blood lymphocytes were determined by quantitative real-time PCR following porcine reproductive and respiratory syndrome virus (PRRSV) restimulation *in vitro*. CTL assay **(H)** was performed with PRRSV-infected porcine alveolar macrophages as target cells and peripheral blood lymphocytes from PRRSV-vaccinated pigs as effector cells. 4-1BB Tg, 4-1BB transgenic pigs; WT, wild-type pigs used as a control. The results shown are representative of three independent experiments. Data are presented as mean values ± SEM. *P* < 0.05 was considered statistically significant.

**Figure 6:**
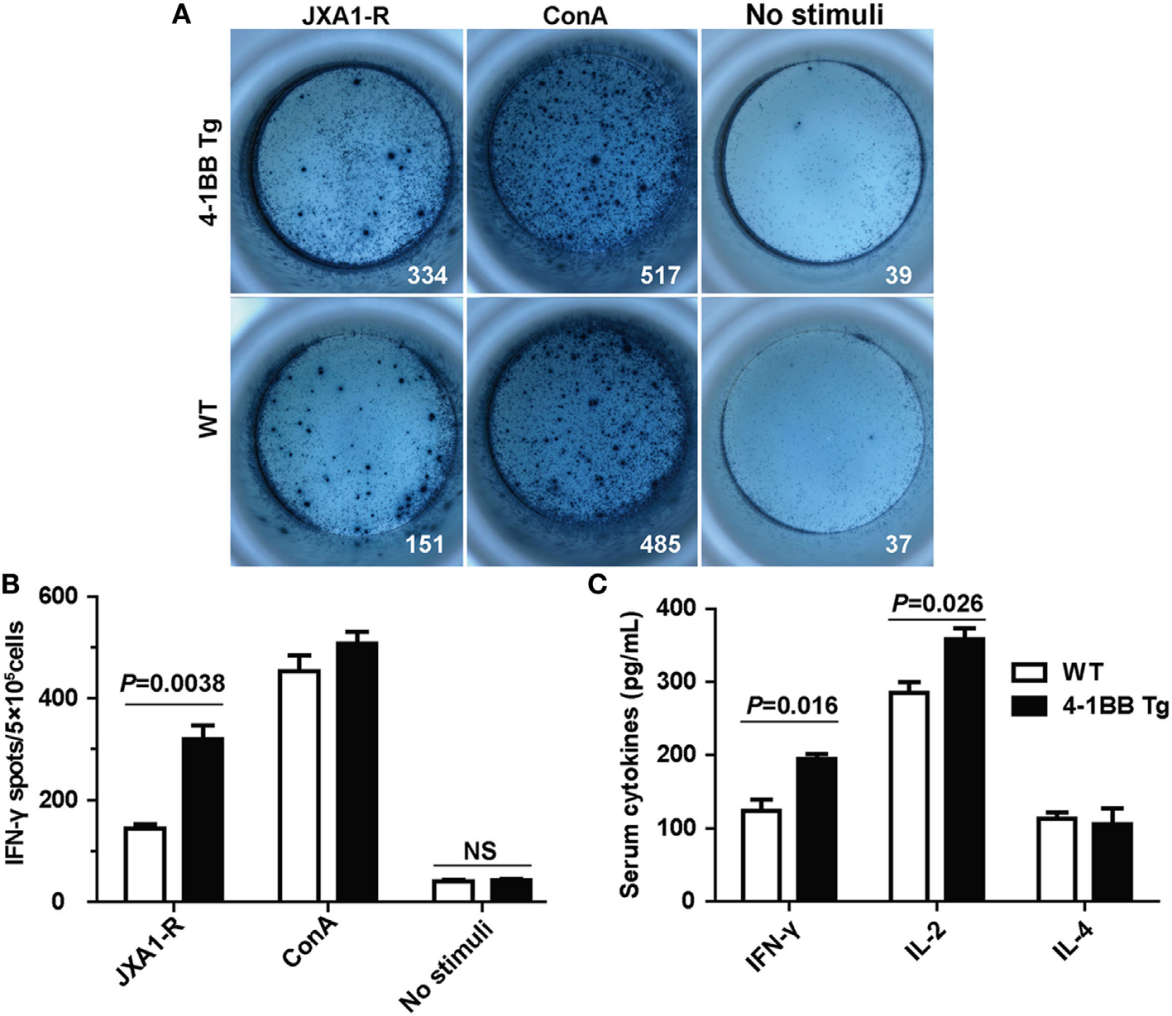
Cytokines secretion by peripheral blood lymphocytes in the Enzyme-Linked ImmunoSpot (ELISPOT) and enzyme-linked immunosorbent assay (ELISA) assays. **(A)** Peripheral blood mononuclear cells (5 × 10^5^ cells/well) from transgenic and wild-type pigs were stimulated with PRRSV JXA1-R or ConA for 36 h. Complete RPMI media were used as the unstimulated controls. Samples were tested in triplicates. Images from the plate scan generated by the ImmunoSpot analyzing system are shown. Average numbers of spots per well are indicated. **(B)** Summary of the spots per well in IFN-γ ELISPOT assay. *P* < 0.05 was considered statistically significant. **(C)** Serum concentrations of IL-2, IFN-γ, and IL-4 were measured by ELISA. Each sample was assayed in triplicate.

We next determined whether co-stimulatory signaling through 4-1BB could augment specific cellular immune response to PRRSV. We found that the IL-2 mRNA expression levels in 4-1BB Tg pigs were nearly threefold higher than those in WT pigs (*P* = 0.016), which indicated that greater T cell expansion occurred in 4-1BB Tg pigs than in WT pigs post *in vitro* stimulation with PRRSV vaccine (Figure 5A). A significant increase in the expression of IFN-γ (*P* = 0.009) and TNF-α (*P* = 0.027), along with a slight decrease in the production of IL-4 (*P* = 0.652), was observed in 4-1BB Tg pigs (Figures 5B–D). This cytokine secretion profile suggested that 4-1BB co-stimulation enhanced the induction of cellular immune responses to PRRSV. We also found that the cytolytic mediator, granzyme B dramatically upregulated in 4-1BB Tg pigs (*P* = 0.008, Figure 5E). No significant differences in the levels of Foxp3 (*P* = 0.082, Figure 5F) and IL-17 (*P* = 0.623, Figure 5G) between Tg and WT pigs. Compared with the WT control, 4-1BB transgenic PBMCs showed a significant increase in lysis of porcine alveolar macrophages at the highest E:T cell ratio (100:1, *P* = 0.0025, Figure 5H), with high levels of lysis observed at an E:T cell ratio of 50:1 (*P* = 0.0046, Figure 5H). Moreover, the production of IFN-γ by PBMCs stimulated with PRRSV was also examined using a standard ELISPOT assay. In accordance with qPCR results, the level of PRRSV-specific IFN-γ secreting cells derived from Tg pig was significantly increased (*P* = 0.0038, Figures 6A,B). The serum concentrations of Th1 cytokines IL-2 (*P* = 0.026) and IFN-γ (*P* = 0.016) in Tg pigs were significantly higher than those of WT control, whereas there is no difference in the level of IL-4, the Th2 cytokine, between both groups (Figure 6C). These data indicate that 4-1BB plays a critical role in CTL-mediated specific anti-PRRSV immune responses.

## Discussion

Co-stimulation mediated by 4-1BB is essential for CD8^+^ T cell proliferation and cytolytic function (46–50). Here, we tested the hypothesis that expression of higher levels of 4-1BB in Tg pigs can enhance their cellular immune responses. By virtue of CRISPR/Cas9-mediated homologous recombination, we generated Tg pigs expressing an extra copy of 4-1BB. Using this model, we found that 4-1BB signaling plays a significant role in Th1 induction, differentiation, and CTL effector function.

In this study, the higher level of 4-1BB expression in Tg pigs led to T cell expansion, as well as an increased level of IL-2 during the primary response, consistent with the findings in 4-1BBL^−/−^ mice (27). IFN-γ production was usually low during PRRSV infection (12, 13). However, it was upregulated in PBMCs of 4-1BB Tg pigs during both the primary and secondary responses, and the CTL effector function was also significantly enhanced in 4-1BB Tg pigs. These data indicate that 4-1BB signaling can enhance cell-mediated antiviral responses.

Previous studies have shown that the formation of antigen-specific antibodies is dependent on CD4^+^ T cells providing help to B cells, with IL-4 being central to this process (51, 52). However, the titer of PRRSV-specific IgG antibody in the serum was not significantly different between 4-1BB Tg and WT pigs, consistent with the production of similar levels of IL-4 in the primary and secondary responses. This means that Th2-type responses are not impacted in 4-1BB Tg pigs.

Cytotoxic T lymphocyte-mediated cellular immunity is essential for the elimination of intracellular pathogens. However, conventional PRRSV vaccines fail to elicit adequate cellular immune responses and therefore cannot efficiently protect vaccinated swine from becoming infected and shedding viruses (53, 54). In this study, CTL proliferation and responsiveness following stimulation with PRRSV antigens *in vitro* were enhanced by amplifying the 4-1BB signal with the insertion of a second gene copy. These results agree with the recognized role of 4-1BB signaling in the CTL response during secondary viral infection (24, 55–57).

Although deletion of CD163, a fusion receptor for PRRSV, has been described resulting in a significant resistance to the virus infection (33, 58), the CD163^−/−^ pigs still have a risk of infection with other pathogens that do not depend on this receptor. It has been proved that the broad-spectrum, immunity-promoting molecule 4-1BB induces T cell activation and survival, as well as CTL induction, which facilitates the clearance of intracellular pathogens (25–27). Tan et al demonstrated that 4-1BB co-stimulation is important in the generation of CTL responses after vaccination, inducing long-term immunity that is protective against viral challenge (59). As shown in our study, an extra copy of the porcine 4-1BB gene specifically integrated into the porcine genome, leading to the enhanced Th1 type cytokines production and CTL responses against PRRSV vaccination and suggesting its promising application for maximizing the effects of current vaccines to prevent infectious diseases in pigs.

Although the impact of 4-1BB-mediated signaling on the immune response to PRRSV vaccination was documented *in vitro*, we did not conduct an *in vivo* challenge assay using vaccinated pigs because of the small number of Tg pigs available and we had to keep the Tg founder pig for propagation. The role of 4-1BB co-stimulation in immune responses to infection with PRRSV and other intracellular pathogens such as bacteria and parasites will also be examined in future studies.

In conclusion, for the first time, we report the generation of 4-1BB Tg pigs with the enhanced co-stimulatory signaling through transgenic technology. The observed effects of 4-1BB on the induction, amplification and persistence of CTL responses suggest the use of this methodology as a novel strategy for increasing the potency of vaccines against infectious diseases. Our study provides a novel concept for breeding farm animals with increased immune responses to vaccination and resistance to a broad spectrum of infectious pathogens through transgene of one or more copies of co-stimulation molecules like 4-1BB into the animal genome.

## Ethics Statement

All animal experiments in this study were performed in strict accordance with the Guide for the Care and Use of Laboratory Animals of the Ministry of Science and Technology of China and were approved by the Institutional Animal Care and Use Committee of China Agricultural University (certificate of the Beijing Laboratory Animal employee, ID: 18086). All efforts were made to minimize animal suffering.

## Author Contributions

The study was conceived and experiments designed by XS, QL, GH, and XL. The experiments were performed by GH, ZL, and XT. Data were analyzed and interpreted by XS, QL, and GH. XS, QL, and ZL provided reagents, materials, or analysis tools. While GH drafted the paper, and XL, DD, E-AS, XS, and QL all revised the manuscript, contributing important intellectual content.

## Conflict of Interest Statement

The authors declare that this research was conducted in the absence of any commercial or financial relationships that could be construed as a conflict of interest.
